# Evaluating the effects of volume censoring on fetal functional connectivity

**DOI:** 10.1038/s41598-025-96538-x

**Published:** 2025-04-16

**Authors:** Jung-Hoon Kim, Josepheen De Asis-Cruz, Kevin M. Cook, Catherine Limperopoulos

**Affiliations:** Developing Brain Institute, Children’s National, 111 Michigan Ave N.W., Washington D.C., 20010 USA

**Keywords:** Fetus, rs-fMRI, Head motion, Motion censoring, Age prediction, Sex prediction, Neuroscience, Paediatric research, Computational neuroscience

## Abstract

Advances in neuroimaging have enabled non-invasive investigation of fetal brain development in vivo. Resting-state functional magnetic resonance imaging (rs-fMRI) has provided critical insights into emerging brain networks in fetuses. However, acquiring high-quality fetal rs-fMRI remains challenging due to the unpredictable and unconstrained motion of the fetal head. Nuisance regression, where the brain signal is regressed onto translational and rotational head motion parameters, has been widely and effectively used in adults to reduce the influence of motion. However, subsequent studies have revealed that associations between head motion and large-scale brain functional connectivity (FC) persisted even after regression. In ex utero groups (e.g., newborns, toddlers, and adults), censoring high-motion volumes has shown effectiveness in mitigating such lingering impacts of head motion. While censoring high motion volumes has been utilized in fetal rs-fMRI, a systematic assessment of the effectiveness of regression and censoring high motion volumes in fetuses has not been done. Establishing the effectiveness of censoring in fetal rs-fMRI is critical to avoid possible bias in findings resulting from head motion. To address this knowledge gap, we investigated the associations between head motion and fetal rs-fMRI at different analysis scales: blood oxygenation level dependent (BOLD) time series and whole-brain FC. We used a dataset of 120 fetal scans collected from 104 healthy fetuses. We found that nuisance regression reduced the association between head motion, defined by frame-by-frame displacement (FD) of head position, and BOLD time series data in all regions of interest (ROI) encompassing the whole brain. Nuisance regression, however, was not effective in reducing the impact of head motion on FC. Fetuses’ FC profiles significantly predicted average FD (*r* = 0.09 ± 0.08; *p* < 10^–3^) after regression, suggesting a lingering effect of motion on whole-brain patterns. To dissociate head motion and the FC, we used volume censoring and evaluated its efficacy in correcting motion at different thresholds. We demonstrated that censored data improved resting state data’s ability to predict neurobiological features, such as gestational age and sex (accuracy = 55.2 ± 2.9% with 1.5 mm vs. 44.6 ± 3.6% with no censoring). Collectively, our results highlight the importance of data censoring in reducing the lingering impact of head motion on fetal rs-fMRI, thus attenuating motion-related bias. Like older age groups such as neonates and adults, combining regression and censoring techniques is recommended for large-scale FC analysis, e.g., network-based analysis, for fetuses.

## Introduction

Resting-state functional magnetic resonance imaging (rs-fMRI) has contributed significantly to our understanding of fetal brain development^1–5^. Despite the potential of fetal rs-fMRI in enhancing our understanding of in utero brain development, concerns persist regarding the reliability and robustness of estimated connectivity due to higher fetal head motion, potentially confounding measured brain signals. In adults, the disruptive influence of head movement during rs-fMRI scans on functional connectivity has been extensively studied^6–8^. For example, it has been shown that head motion can alter the strength of functional connectivity (FC), possibly distorting the pattern of large-scale brain networks, e.g., default mode network (DMN)^8^. Furthermore, the adverse impact of head motion is evident in case–control studies, where head motion introduces artifactual group-wise differences (subjects with low vs. high motion) in brain network patterns^7–10^. Similar to adults, the confounding effects of head motion have also been described in younger age groups, including toddlers and infants^11^, and even newborns^12^. In the fetal population, correcting for the effects of head motion is even more challenging as there are no existing means to limit fetal head motion during scans, potentially compromising the reliability of rs-fMRI and functional connectivity measures. Yet, the impact of head motion on fetal rs-fMRI remains largely unstudied.

To address the impact of head motion on rs-fMRI data, several motion correction techniques have been proposed and extensively validated using large-scale rs-fMRI datasets^7,13–15^. Nuisance regression combined with censoring of high motion volumes is a commonly used approach for effectively minimizing the influence of head motion on measured blood-oxygenation-level-dependent (BOLD) signals. In the early days, regressing the BOLD signal onto translational and rotational head motion parameters (and its derivatives) was used alone to correct motion^16^. However, associations between head movement and FC persisted after regression^7,8,17^. Later studies reported that volume censoring, where frames with high motion were excluded from the analysis^7^, further minimized motion effects. The effectiveness of nuisance regression + censoring (Reg + Cen) in attenuating the influence of head motion on rs-fMRI has been demonstrated and validated in ex utero populations, from neonates to adults^7,11,12,18,19^. Taymourtash et al.^20^ demonstrated the utility of nuisance regression in reducing the impact of head motion on fetal FC. Recently, censoring has been utilized in fetal rs-fMRI studies, including out sides^3,21–24^. As such, in the fetal rs-fMRI field, there have been efforts to propose the state-of-art preprocessing guidance, e.g., RS-FetMRI pipeline—semi-automatic standardized fetal rs-fMRI preprocessing pipeline developed by Pecco et al., (https://github.com/NicoloPecco/RS-FetMRI)^25^. However, the benefits of censoring in fetal rs-fMRI have not been systematically explored. Our study attempts to address this critical gap.

In this study, we hypothesized that: (1) fMRI data with only nuisance regression retains noise originating from head motion; (2) fMRI volume censoring at an optimal motion threshold alleviates this lingering motion effect; and (3) applying censoring to fMRI data improves the signal-to-noise ratio (SNR), enhancing prediction accuracy for neurobiological features in fetuses. To rigorously evaluate the effect of head motion on fMRI data, we applied machine-learning algorithms to predict motion or neurobiological features using censored fMRI data. We investigated the impact of head motion on fetal resting-state fMRI using 120 fetal scans acquired at Children’s National in Washington, DC. Our findings collectively support the use of data censoring alongside motion regression as a preprocessing step in fetal resting-state fMRI, consistent with recommendations for other age groups. Importantly, we showed that censoring data at an appropriate threshold improved the prediction accuracy of neurobiological features, such as age and biological sex.

## Materials and methods

### Subjects

Pregnant women with low-risk pregnancies were recruited as part of a longitudinal project investigating brain development in complex congenital heart disease at Children’s National Hospital in Washington, DC. In total, 120 rs-fMRI scans from 104 healthy fetuses were analyzed. Sixteen fetuses had two scans. All experiments were conducted under the regulations and guidelines approved by the Institutional Review Board (IRB) of Children’s National; written informed consent was obtained from each pregnant woman who participated in the study. Only fetuses with structurally normal brains were included in the study. Pregnant women with psychiatric/metabolic/genetic disorders, complicated pregnancies (i.e., preeclampsia and gestational diabetes), multiple pregnancies, maternal medications, and contraindications to MRI were excluded from the study. Fetal exclusion criteria were dysmorphic features by antenatal ultrasound, chromosomal abnormalities by amniocentesis, presentation after 28 weeks gestational age, multiple gestations, and evidence of congenital infections.

### MRI acquisition

Structural and functional resting-state MR images (sMRI and rs-fMRI, respectively) were acquired using a 1.5 Tesla GE MRI scanner with an 8-channel receiver coil. T2-weighted images (i.e., sMRI) were collected using the following settings: single-shot fast spin-echo sequence, TR = 1100 ms, TE = 160 ms, flip angle = 90 degree, and voxel size = 0.8 × 0.8 × 2 mm. Blood-oxygen-level-dependent (BOLD) signal, i.e., rs-fMRI activity, was acquired using echo planar imaging sequence with the following settings: TR = 3000 ms, TE = 60 ms, flip angle = 90 degrees, field of view = 33 cm square in-plane, matrix size = 128 × 128 (# of z-axis slices varied across subjects to ensure the full coverage of fetal head), and voxel size = 2.58 $\times$ 2.58 $\times$ 3 mm. We acquired 144 volumes (~ 7 min) per rs-fMRI scan, except for two scans with 104 volumes (~ 5 min).

### Preprocessing of fetal RS-fMRI data

We used a previously validated pipeline to preprocess the rs-fMRI datasets^3,26^. The preprocessing steps were implemented using irtk^27^, AFNI^28^, Bioimage Suite^29^, and in-house MATLAB code. The following preprocessing steps were applied: (1) re-orientation of images, (2) within-volume realignment, (3) de-spiking, (4) bias-field correction, (5) slice time correction, (6) motion correction^29,30^, (7) co-registration of fMRI to T2 anatomical MR (using *flirt* from FSL), (8) intensity scaling^31^, and (9) spatial smoothing at full-width-half-maximum = 4.5 mm. Note that de-spiking step was implemented using *3dDespike* from *AFNI* and functioned to replace outlier volumes with smoothed values; different from volume censoring (for details, see *3dDespike* from *AFNI).* For the motion correction step, we utilized *fetalmotioncorrection* function from Bioimage Suite*.* Specifically, successive image volumes are co-registered to a reference volume using rigid body transformation, and the reference volume was set to the volume with the lowest outlier fraction (estimated using *3dToutcount* from AFNI). During the motion correction step, motion regressors were calculated. Different number of motion regressors were employed, # of regressors = 6, 12, 24, and 36, which have been widely used in previous studies^19,32,33^. Six motion parameters $\text{R}$ consisted of three translational and three rotational parameters: [X Y Z pitch yaw roll]. For 12, regressors included their first-order derivates [${\text{R}}$ $R^{\prime}$]. The 24 and 36 regressors were derived by Volterra expansion: 24 regressors consisted of [$\text{R}$ ${\text{R}}^{2}$ ${\text{R}}_{\text{t}-1}$ ${\text{R}}_{\text{t}-1}^{2}$], where t and t − 1 refer to the current and immediately preceding time point, and 36 regressors consisted of [$\text{R}$ ${\text{R}}^{2}$ ${\text{R}}_{\text{t}-1}$ ${\text{R}}_{\text{t}-1}^{2}$ ${\text{R}}_{\text{t}-2}$ ${\text{R}}_{\text{t}-2}^{2}$]. Among different sets, 12 regressors were used as the default unless otherwise specified. Rotational parameters defined in radians were converted to millimeters given the estimated radius of individual fetal brains. On top of motion-related regressors, noise parameters included the first three principal components of signals from white matter and ventricles^34,35^. White matter and ventricles were identified using the deep learning-based segmentation algorithm^36^ and tissue masks were finalized after manual inspection. Finally, using calculated regressors, censoring (if applied), bandpass filtering at 0.01–0.1 Hz, and nuisance regressions were applied simultaneously. Steps implemented in our fetal preprocessing pipeline are largely consistent with preprocessing pipelines established by other research groups, including Pecco et al.^25^, enhancing the robustness of our preprocessing pipeline.

Fetal brains were functionally parcellated into 200 regions of interest (ROI) covering the cerebrum, cerebellum, and brainstem using a spectral clustering algorithm^37^. For each ROI, we computed its (1) region-wise BOLD fluctuations, or the averaged BOLD time series data across all voxels within each ROI, (2) individual FC, the Pearson correlation between a pair of ROIs, and then converting them to Fisher’s *z*-score and, (3) its functional connectivity (FC) profile, the collection of individual FCs across all ROI pairs. The FC profile consisted of all ROI-to-ROI FC for the whole brain (# of FCs $=\frac{200\times (200-1)}{2} =\text{19,900}$, representing all possible pairwise combinations for 200 ROIs).

### Framewise displacement

Per volume (or frame) translational (x-, y-, and z-axis) and rotational (roll, pitch, and yaw) head motion parameters were obtained during the motion correction step described above. Rotational parameters defined in radians were converted to millimeter scale. For *i*^th^ frame, frame-wise displacement (${FD}_{i}$) was computed by measuring the head displacement. Formerly, ${FD}_{i}$ was defined as

$${FD}_{i}= \left|\Delta {d}_{ix}\right|+ \left|\Delta {d}_{iy}\right|+ \left|\Delta {d}_{iz}\right|+ \left|\Delta {\phi }_{i}\right|+ \left|\Delta {\theta }_{i}\right|+ \left|\Delta {\psi }_{i}\right|,$$

Where $\Delta {d}_{ix}= {d}_{(i-1)x}- {d}_{ix}$, or the positional displacement at the x-axis from the $i-1$ to *i*th frame, and similarly for ${d}_{iy}$, ${d}_{iz}$, $\Delta {\phi }_{i}$, $\Delta {\theta }_{i}$, and $\Delta {\psi }_{i}$ (y, z, roll, pitch, and yaw, respectively). ${FD}_{0}$ was set to 0. For each scan, we calculated the average FD (FD_ave_), representing the mean FD across the preserved volumes, and the maximum FD (FD_max_), indicating the highest FD value among the remaining volumes.

### Data censoring

We investigated the associations between head motion and rs-fMRI data by correlating head motion with (1) ROI-based BOLD fluctuations (i.e., the timecosurse of voxels averaged within each ROI), (2) individual FC, and (3) FC profiles (defined above). Rs-fMRI data sets were uncensored (i.e., Reg only or full time series) or censored (Reg + Cen); volumes were censored at different FD levels: 0.5, 1.0, 1.5, 2.0, and 2.5 mm. These thresholds were chosen based on commonly used threshold levels in adults^7,33,38^, newborns^12^, and fetuses^22^. Here, censoring was conducted by removing corrupted volumes and temporally concatenating data back together again. Note that the censoring, if applied, was applied simultaneously with nuisance regression and bandpass filtering, to prevent leakage of high-motion volume to neighboring volumes. As regression, filtering, and censoring procedures were applied simultaneously, the degrees of freedom of data were proportional to the time points of censored fMRI. After censoring fMRI scans based on the threshold level, FD_ave_ and FD_max_ were calculated.

### Temporal signal-to-noise (tSNR) ratio

We assessed the quality of fetal fMRI data by computing the temporal signal-to-noise ratio (tSNR)^39,40^ on the bias-field corrected volumes. Previous studies have suggested that tSNR is sensitive to fMRI activation^41^ and statistical detection power^42^. For *i*th voxel, tSNR was defined as

$$tSNR_{i} = \frac{{\overline{S}_{i} }}{{\sigma_{i} }},$$

where ${\overline{S} }_{i}$ and ${\sigma }_{i}$ are the mean and standard deviation, respectively, of the BOLD signal over timeseries at the *i*th voxel. Once calculated, the tSNR measure was averaged over voxels within the brain mask to yield the representative signal quality of an individual’s fMRI data.

### Head motion and GA

To investigate whether head motion was related to advancing gestational age (GA), we correlated GA with FD_ave_ and FD_max_ across different threshold levels using censored and uncensored data. False-discovery-rate (FDR) correction was applied for multiple comparison correction.

### Head motion and region-wise BOLD fluctuations

To determine if Reg+Cen reduced residual effects of motion on the BOLD data, we correlated FD and region-wise BOLD fluctuations using Pearson correlation, yielding correlation coefficient (*r*), and p-value. We then averaged the squared correlation coefficient (*r*^2^) across different ROIs or subjects. The correlation was squared as the association between FD and regional BOLD could be positive or negative (see Fig. 3).* P*-values of each correlation were tested at different statistical threshold levels (uncorrected *p*, *p*_*unc*_ < 0.05, *p*_*unc*_ < 0.01, or FDR-corrected *p*, *p*_*FDR*_ < 0.05). We further tested the association between FD and BOLD data with the limited data size (# = 40 volumes).

### Association of head motion to the strength of individual FCs

We calculated the Pearson correlation between FC strength and both FD_ave_ and FD_max_. Pearson’s correlation was converted to Fisher’s z-score. Here, conversion to an accurate z-score was conducted using the “xDF” method^43^, which can consider the heterogenous fMRI autocorrelation properties due to different scan lengths. Then, we explored the FD-FC association across all 19,900 connections for the group. We presented results at different statistical thresholds (*p*_*unc*_ < 0.05, *p*_*unc*_ < 0.01, or *p*_*FDR*_ < 0.05). We further investigated the relationship between the strength of FCs and anatomical distances of FCs. Distance between ROIs was defined as Euclidean distance between centroids of ROIs.

### Head motion and FC profile

In our fMRI preprocessing pipeline, similar to conventional approaches, we applied nuisance regression to our data, effectively minimizing head motion’s influence on fMRI time courses and individual FC. In older age groups, such as neonates and adults, head motion’s influence on FC profile persisted despite successful motion regression^7,12,44–48^. To test it, we built a model that compared fetal FC profiles’ ability to predict non-neural versus neurobiological variables. Again, the FC profiles were constructed from uncensored or censored data at different FD thresholds. Higher prediction accuracy for non-neural outcomes, in this case FD_ave_ and FD_max_, would suggest that the motion correction approach and threshold did not effectively minimize the impact of motion. An effective method should demonstrate improved predictability for neurobiological features. In this study, we selected GA and biological sex as target features. This decision was based on findings from our previous studies^3,22^, which showed differing levels of difficulty in FC-based prediction of GA versus biological sex. We anticipated that evaluating changes in prediction accuracy using multiple neurobiological features (GA and sex) would provide a more robust range of threshold levels compared to using just a single feature, whether GA or sex.

Using a five-fold hold-out cross validation (CV) method, we tested how well FC profiles predicted GA, FD, and biological sex. As censoring at the level of 0.5 mm yielded limited data length, dataset censored at 0.5 mm was not used in this analysis. We split the data into training (= 96, 80% of 120 scans) and test (= 24, 20% of 120 scans) sets. Using the training scans, we chose connections (i.e., feature selection) that were significantly associated with these three variables (*p*_*unc*_ < 0.05). To assess significance, we used Pearson correlation for continuous data –FD_ave_, FD_max_, and GA—and two-sample t-test for biological sex, as illustrated in Fig. 5A. To train the model, support vector regression (SVR) or classification (SVC) were implemented using the MATLAB code *fitrsvm.m* for FD_ave_, FD_max_, and gestational age and *fitsvm.m* for biological sex. The default parameters provided by MATLAB were used for both SVR and SVC models. We then validated the model on the test set. To assess prediction accuracy for the continuous variables, in the test/held-out validation dataset, we measured the Pearson correlation between the actual versus predicted values of FD_ave_ or FD_max_ and GA. Pearson correlation was converted to z-score using Fisher’s z-transform. For biological sex, the prediction accuracy was the percentage of scans correctly predicted in the held-out validation dataset. To improve the reliability of measured prediction performance and ensure no data leakage between training and testing sets, we utilized nested cross-validation, repeating the cross-validation analysis 50 times, each time assigning scans to the training and testing datasets differently. Fisher’s z-transformed *r* (for continuous variables) and accuracy (for biological sex) were averaged over 50 cross-validation trials. The statistical significance of prediction performance was tested using a permutation test. The null prediction performances were generated from 50 permutation sets. For each permutation set, variables were randomly shuffled/assigned, and the prediction performance of this set was calculated using the identical cross-validation scheme with shuffled variables. A *p*-value was calculated using one-tailed t-test (original > random). If the *p*-value was below 0.05, the result was considered statistically significant.

### Whole brain connectivity of the motor network

Finally, we investigated the impact of head motion on large-scale networks, specifically the motor network. To define the motor network for the group, we warped all fMRI scans onto a spatiotemporal fetal brain atlas^49^. To minimize the error during the alignment procedure, individual scans were warped to a brain atlas in two steps: (1) warp to an age-matched CRL template, (2) and re-warped to 32 weeks CRL brain template. For each scan, using the right precentral region (Fig. 6A) as the seed region of seed-based correlational analysis, we estimated a connectivity strength map reflecting the correlation between fMRI activity at the seed region and fMRI fluctuations over the whole brain. Individual Pearson correlation maps were transformed into Fisher’s z-score maps and averaged.

Next, we examined the effect of head motion on whole brain connectivity of the motor network by contrasting statistical parametric maps of low- versus high-motion groups. Each subgroup comprised 10% of the scans (~ 12 out of 120 data sets). The top 10% with the lowest mean FD were categorized as the low-motion group and vice versa for the higher-motion group. As mean FD was measured after censoring, groups with low/high motion were defined for each censoring condition.

The significance of within-group connectivity patterns was tested using a one-sample t-test with clustering-extent-based threshold (min cluster # = 40 with uncorrected *p* < 0.01). The group-wise difference in connectivity patterns was tested using a two-sample t-test with the same cluster-based statistical threshold. The size of the group-level effect of head motion was evaluated by counting the number of significantly different voxels between high- vs. low-motion groups and individual clusters.

### Complementary measure for head motion on volumes

In addition to FD, we incorporated an additional measure of head motion, DVARS—the spatial root mean square of the data following temporal differencing. Specifically, scaled DVARS (sDVARS) was calculated using the AFNI function 3dTto1D. The DVARS values were derived from voxels within the gray matter and rescaled by their grand mean of intensity (over voxel) to ensure comparability across subjects. We then evaluated sensitivity and specificity across varying sDVARS thresholds relative to the FD threshold of 1.5 mm. Finally, we analyzed the prediction accuracy of neurobiological features, including age and sex, using fMRI data censored by either sDVARS alone or a combination of FD and sDVARS.

## Results

### Subjects

Fetal GAs ranged between 19.14 and 39.70 weeks (mean ± SD = 33.59 ± 4.05; median: 34.95; 25 and 75 IQR: 31.30 and 36.40). There were 50 males and 54 females in the sample. For the 16 fetuses with two scans, the second scan was performed at least three weeks after the first visit. The interval between scans ranged from three to 12.9 gestational weeks (mean and SD: 7.4 ± 2.7).

### The effect of censoring strategy on fMRI data

**Fig. 1:**
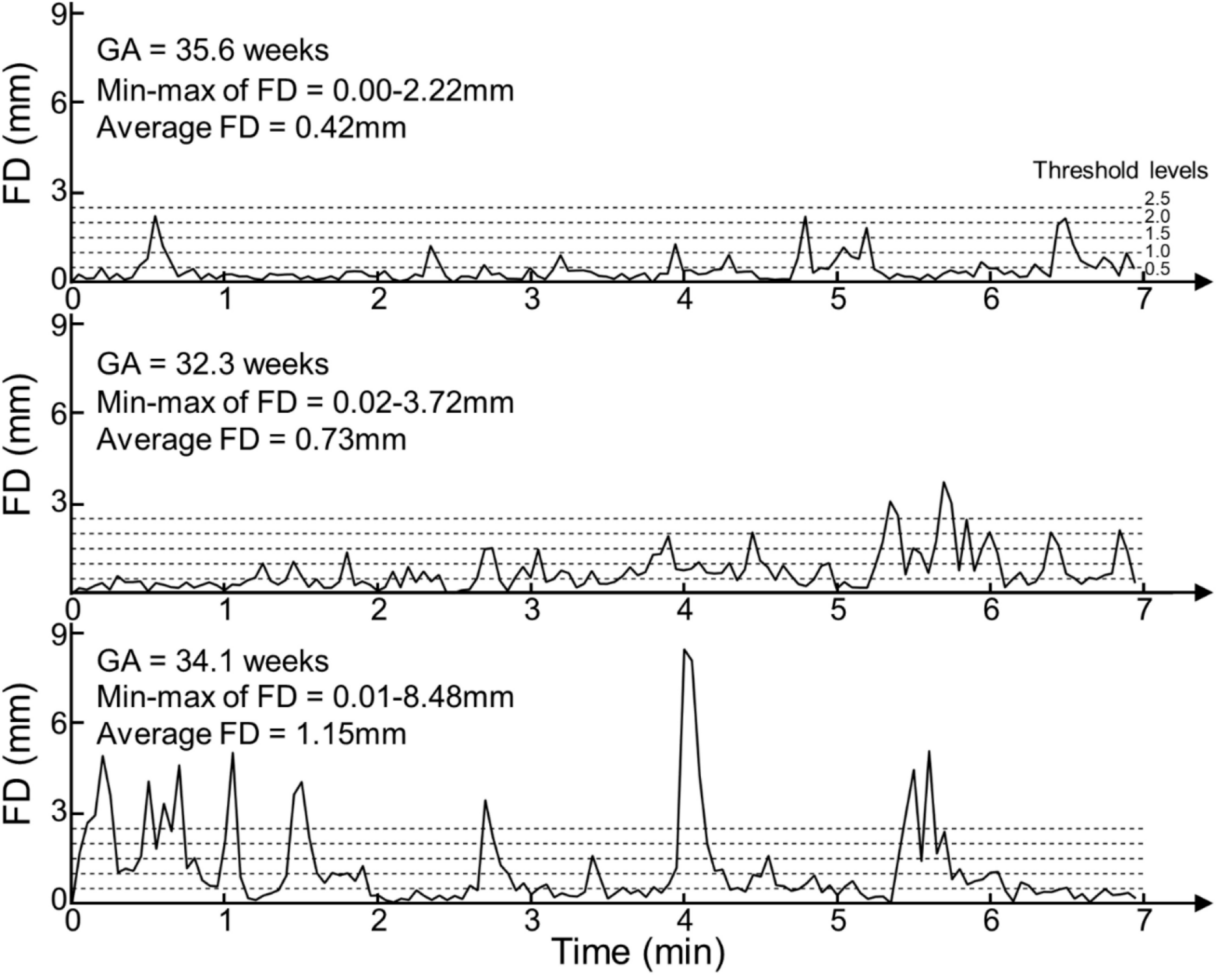
**Frame-wise displacement (FD) over time**. Motion fluctuation during scans in 3 representative fetuses: low, moderate, and high motion shown in the top, mid, and bottom panels, respectively. Scans exhibited periods of low head motion (< 0.5 mm) interspersed with shorter intervals of higher head movement (e.g., > 1.5 mm). The frequency and intensity of these intermittent high-motion periods varied among scans. For instance, head motion spikes (> 1.5 mm) in the low-motion scan (top panel) occurred only four times (~ 3.6% of scans) compared to more than ten times (~ 22.1% of scans) in the high-motion scan (bottom panel). The average and standard deviation of FD for low-, moderate-, and high-motion scans were 0.42 mm ± 0.42 mm, 0.72 ± 0.65, and 1.15 ± 1.45, respectively.

Head motion traces for low, medium, and high motion scans are shown in Fig. 1. FD_ave_ and FD_max_ values for uncensored and censored rsfMRI are shown in Table 1. After censoring, FD_max_ decreased by 25.3, 20.9, 16.3, 11.5, and 5.6% for threshold levels of 2.5, 2.0, 1.5, 1.0, and 0.5 mm, respectively. As expected, censoring with stricter threshold levels made the data more discontinuous, leading to a shorter length of each data chunk (Table 2).

**Table 1:** Distribution of frame-wise displacement at different censoring levels.

Threshold level	FD_ave_				FD_max_			
	Min–Max	Mean	Median	SD	Min–Max	Mean	Median	SD
No Censor	0.27–2.15	0.91	0.83	0.42	0.90–80.08	8.29	5.90	8.83
2.5 mm	0.27–0.91	0.59	0.60	0.14	0.87–2.49	2.10	2.22	0.39
2.0 mm	0.27–0.84	0.56	0.57	0.13	0.87–2.00	1.73	1.81	0.26
1.5 mm	0.23–0.80	0.52	0.51	0.12	0.84–1.50	1.35	1.42	1.60
1.0 mm	0.20–0.68	0.45	0.45	0.09	0.55–1.00	0.95	0.97	0.06
0.5 mm	0.16–0.44	0.29	0.29	0.05	0.23–0.50	0.47	0.48	0.04

*FD* Frame-wise displacement, *SD* Standard Deviation. Unit: mm

**Table 2:** Effect of volume censoring on data continuity.

Threshold level (mm)	# of Chunk (min–max)	Data length of chunk (TRs; min–max)
2.5	3.3 (1–8)	56.6 (11.5–140)
2.0	4.0 (1–8)	43.8 (10.8–140)
1.5	5.2 (1–12)	32.1 (6.6–140)
1.0	8.0 (1–16)	17.2 (2.5–140)
0.5	7.6 (1–17)	4.2 (1–24.3)

**Fig. 2:**
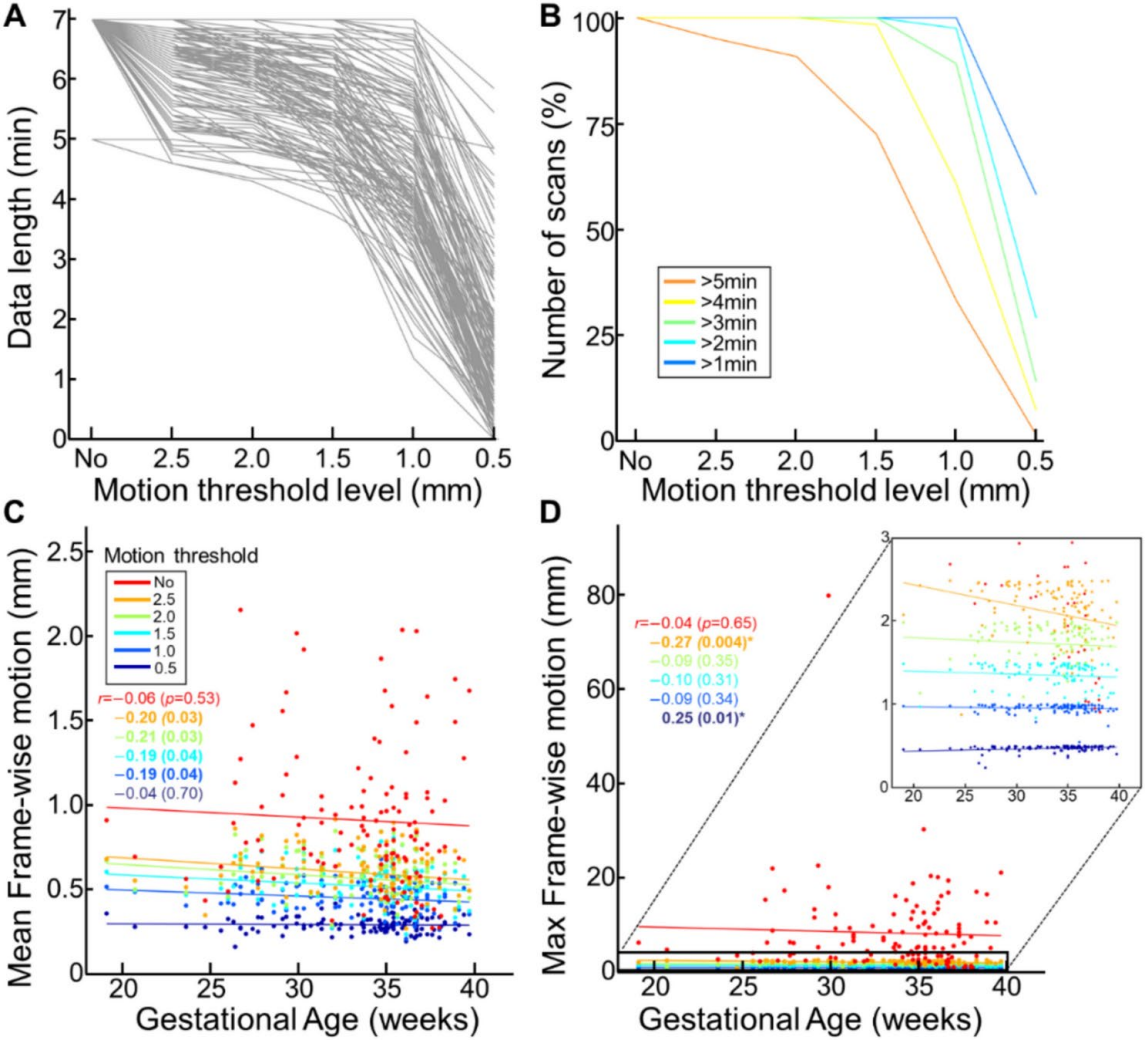
**Frame-wise displacement (FD) in fetuses.** (**A**) Available brain volumes decrease at stricter thresholds: without censoring (= 140 vols ~ 7 min) > 0.5 mm > 0.2 mm; light gray line = scan volumes per subject. (**B**) Percentage of scans of a particular duration available at different censoring thresholds. (**C**, **D**) The change of FD level (**C**: mean FD; **D**: maximum FD) over increasing GA; line = line of best fit. Uncorrected *p*-values. *: Significant after FDR-correction.

As expected, the data length decreased with more stringent threshold levels (Fig. 2A). At the most stringent censoring threshold of 0.5 mm, some scans had no preserved volumes. Relatedly, fewer scans were available at more stringent thresholds (Fig. 2B). We observed a sharp decrease in the proportion of scans with more than 5 min of preserved data as stricter motion thresholds were applied (orange line in Fig. 2B). More than half of the scans still had data exceeding 1 min (blue line) at the most stringent threshold level of 0.5 mm.

We investigated the correlation between gestational age (GA) and mean FD at different threshold levels (Fig. 2C). There was no significant correlation between GA and FD_ave_ for either uncensored or censored data at any level after multiple comparison correction (*r* = − 0.06, − 0.20, − 0.21, − 0.19, − 0.19, and − 0.04; uncorrected *p* = 0.53, 0.03, 0.03, 0.04, 0.04, 0.70; ranging from no censoring to 0.5 mm; FDR-corrected *p* values > 0.05 for all). In contrast to FD_ave_, FD_max_ showed a significant correlation with GA when high-motion volumes were censored at the threshold levels of 2.5 mm and 0.5 mm (Fig. 2D; 2.5 mm, *r* = − 0.27, FDR-corrected *p* = 0.02; 0.5 mm, *r* = 0.25, FDR-corrected *p* = 0.02). We speculate the negative association between GA and FD_max_ at 2.5 mm was due to the smaller space left in the womb for movement as the fetus gets bigger. It is, however, unclear what drove the positive association at 0.5 mm (1.58 $\pm$ 1.31 min; min–max: 0–5.85).

**Fig. 3:**
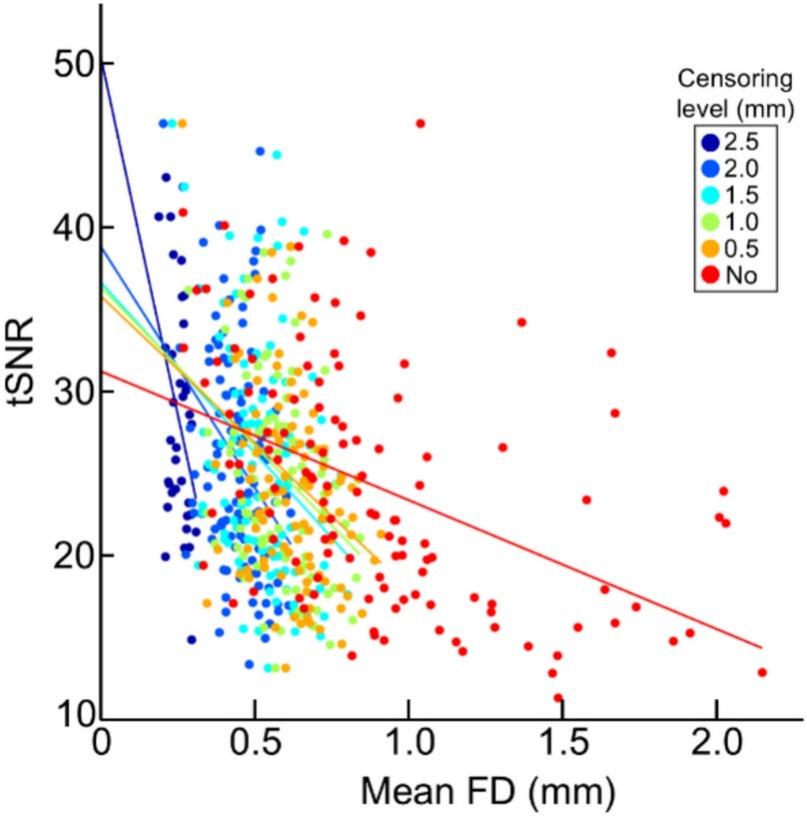
**TSNR decreases with increases in FD.** Lines represent the best fit at each threshold. All *r* scores are significant, *p* < 10^–4^.

Lastly, we investigated the effect of head motion on the tSNR level of fMRI data. Like newborns^12^ and adults^8^, higher head motion in fetuses significantly correlated with lower tSNR (Fig. 3). We further observed that data censoring effectively reduced the association between degree of head motion and TSNR (*r* = − 0.45 for no censoring vs. *r* = − 0.35 and − 0.35 for 1.0 and 1.5 mm, respectively).

### Association between FD and region-wise BOLD fluctuations

**Fig. 4:**
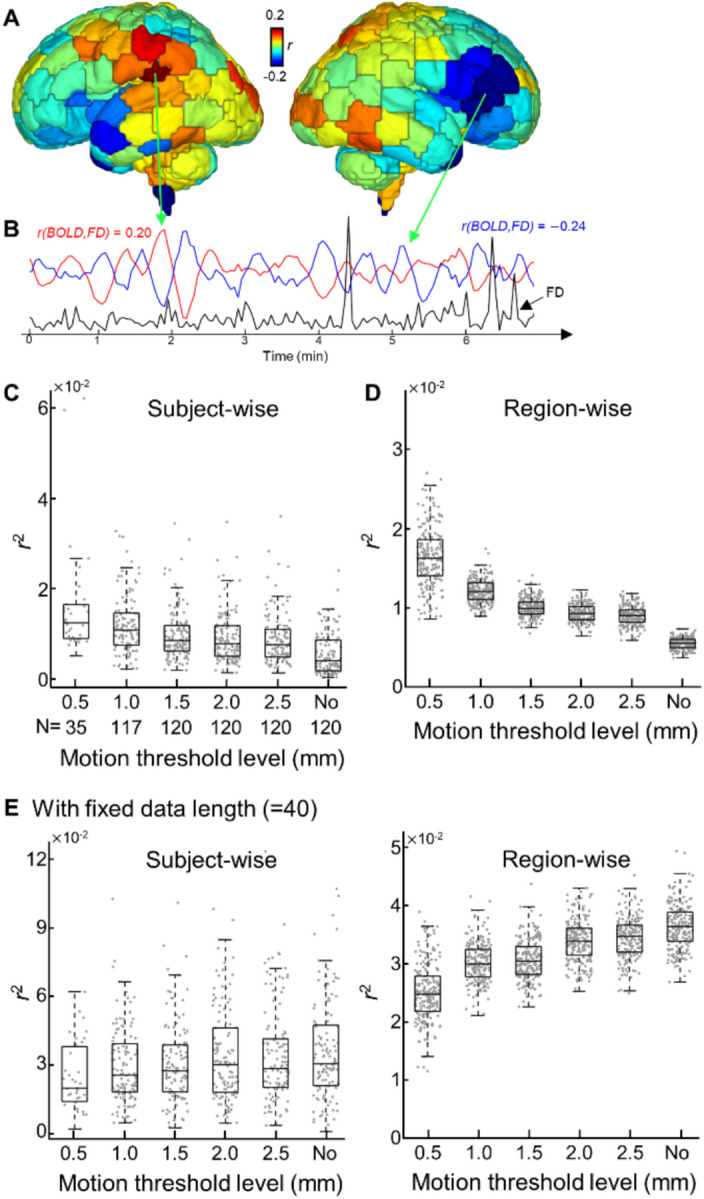
**The effect of head motion on fMRI timeseries.** (**A**) Cortical mapping of association between frame-wise displacement (FD) and fMRI activity, from one representative subject. (**B**) Traces of fMRI activity from ROIs (red or blue lines) and fluctuations of head motion (black line) over the recording period. (**C**, **D**) Boxplot of squared correlation coefficients (*r*^2^) between the trace of head motion and timeseries of ROI. *r*^2^ values are averaged over ROIs (**C**) or subjects (**D**). Each dot represents either each subject (**C**) or each ROI (**D**). N: Number of subjects with 40 volumes that survived different motion-based censoring levels. (**E**) Same as (**C**, **D**) but with the fixed data length across all threshold levels.

**Fig. 5:**
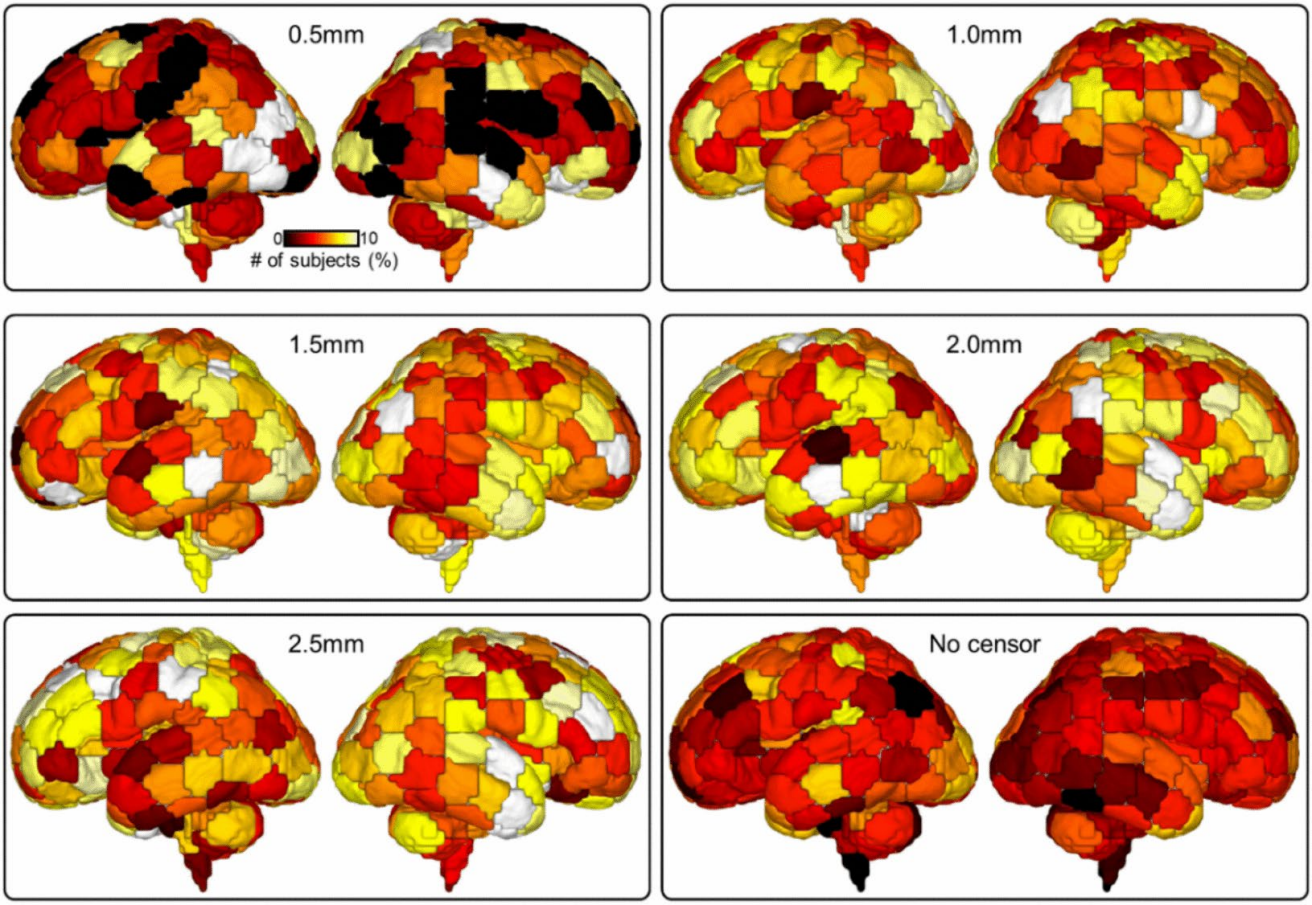
**Cortical mapping of significant associations region-wise BOLD fluctuations and FD.** The percentage of subjects having BOLD-FD association at uncorrected *p* < 0.05 across different threshold levels and without censoring.

We examined the relationship between FD and region-wise BOLD fluctuations over time across the whole brain (Fig. 4A and B). The impact of the censoring strategy was demonstrated by assessing the association (*r*^2^) between FD and BOLD signals per ROI. Here, we analyzed scans with at least two minutes (= 40 volumes) of data remaining after censoring. The number of scans analyzed for each threshold level can be found in Fig. 4C, with the fewest scans (= 35) analyzed for the threshold 0.5 mm. Regardless of the motion threshold level, the association between FD and region-wise BOLD fluctuations post nuisance regression was minimal. Both mean subject-wise *r*^2^ and mean region-wise *r*^2^ ranged between 0.016 to 0.005 for 0.05 mm to no censoring, respectively. Unexpectedly, we observed a decreasing trend of within-group-averaged *r*^2^ (i.e., six samples from 6 different threshold levels) as the motion threshold level became less stringent (Fig. 4C; *r* = − 0.95, *p* = 0.004). Region-wise associations also displayed a decreasing pattern (Fig. 4D; *r* = − 0.95, *p* = 0.004). We speculated this trend was due to the reduced data length with more stringent threshold levels (Fig. 2B). We tested this hypothesis and controlled for data length across different thresholds, i.e., using only 40 timepoints = 2 min. We observed an increasing association between motion and BOLD fluctuations (i.e., higher *r*^2^ values) with less stringent threshold levels (Fig. 4E; *r* = 0.96 and 0.96, *p* = 0.002 and 0.002 for subject-wise and region-wise, respectively). Finally, we tested the significance of these associations (e.g., # of associations = # of subjects × # of ROIs = 120 × 200 in no censoring and = 35 × 200 with a threshold level of 0.05 mm) (Table 3). About 5% of associations were significant (minimum: 3.73% for no censoring; maximum: 6.23% for 2.0 mm; *p*_unc_ < 0.05). We further mapped the number of significant associations (*p*_unc_ < 0.05) to cortical space and found, that while it was relatively distributed uniformly, occipital, temporal, and frontal regions showed more significant associations (Fig. 5). When multiple comparison correction was applied, however, only associations for the 2.5 mm threshold level (0.03%) remained significant (vs. 6.23% at *p*_unc_ < 0.05). These results suggest that motion parameter-based motion-regression strategy (Reg only) effectively removed the influence of head motion on region-wise BOLD fluctuations.

**Table 3:** Associations between region-wise BOLD fluctuations and FD at the level of individual subjects.

Threshold levels	# of significant connections (%)		
	Uncorrected *p* < 0.05	Uncorrected *p* < 0.01	FDR-corrected *p* < 0.05
No censor	3.73	0.88	0
2.5 mm	6.23	1.71	0.03
2.0 mm	6.23	1.63	0
1.5 mm	6.00	1.44	0
1.0 mm	5.46	1.27	0
0.5 mm	4.70	0.83	0

### Association of head motion to the strength of individual FCs

We examined the impact of head motion on pairwise FC. In rs-fMRI data, FC between ROIs is commonly evaluated. If FC is confounded by head motion, subsequent analyses would likewise be affected. For FD_ave_, using a statistical threshold of *p*_*unc*_ < 0.05, approximately 5% of connections, i.e., FC, (minimum: 3.93% with 0.5 mm, maximum: 4.93% with 1.0 mm) were significantly associated with head motion (Table 4). When using a more stringent threshold of *p*_*unc*_ < 0.01, about 1% of connections were significant; no FD_ave_-FC associations remained significant after multiple comparison correction (*p*_*FDR*_ < 0.05). In contrast, censoring at the level of 0.5 mm resulted in the highest percentage of FD_max_-FC associations (with *p*_*unc*_ < 0.05, 8.16% vs. 4–6% at other threshold levels; with *p* < 0.01, 1.96% vs. ~ 1% in other threshold levels). With no censoring or censoring at the level of 1.0 mm, very few FD_max_-FC associations (= 0.01%) were highlighted after the FDR correction.

**Table 4:** Associations between FC and FD at the level of individual subjects.

Threshold level	Average FD (% of connections)			Max FD (% of connections)		
	Uncorrected *p* < 0.05	Uncorrected *p* < 0.01	FDR-corrected *p* < 0.05	Uncorrected *p* < 0.05	Uncorrected *p* < 0.01	FDR-corrected *p* < 0.05
No censor	4.87	1.09	0	4.43	0.97	0.01
2.5 mm	4.59	0.90	0	5.04	0.99	0
2.0 mm	4.44	0.89	0	5.58	1.08	0
1.5 mm	4.63	0.97	0	4.86	1.04	0
1.0 mm	4.93	0.94	0	5.79	1.40	0.01
0.5 mm	3.93	0.70	0	8.16	1.96	0

**Fig. 6:**
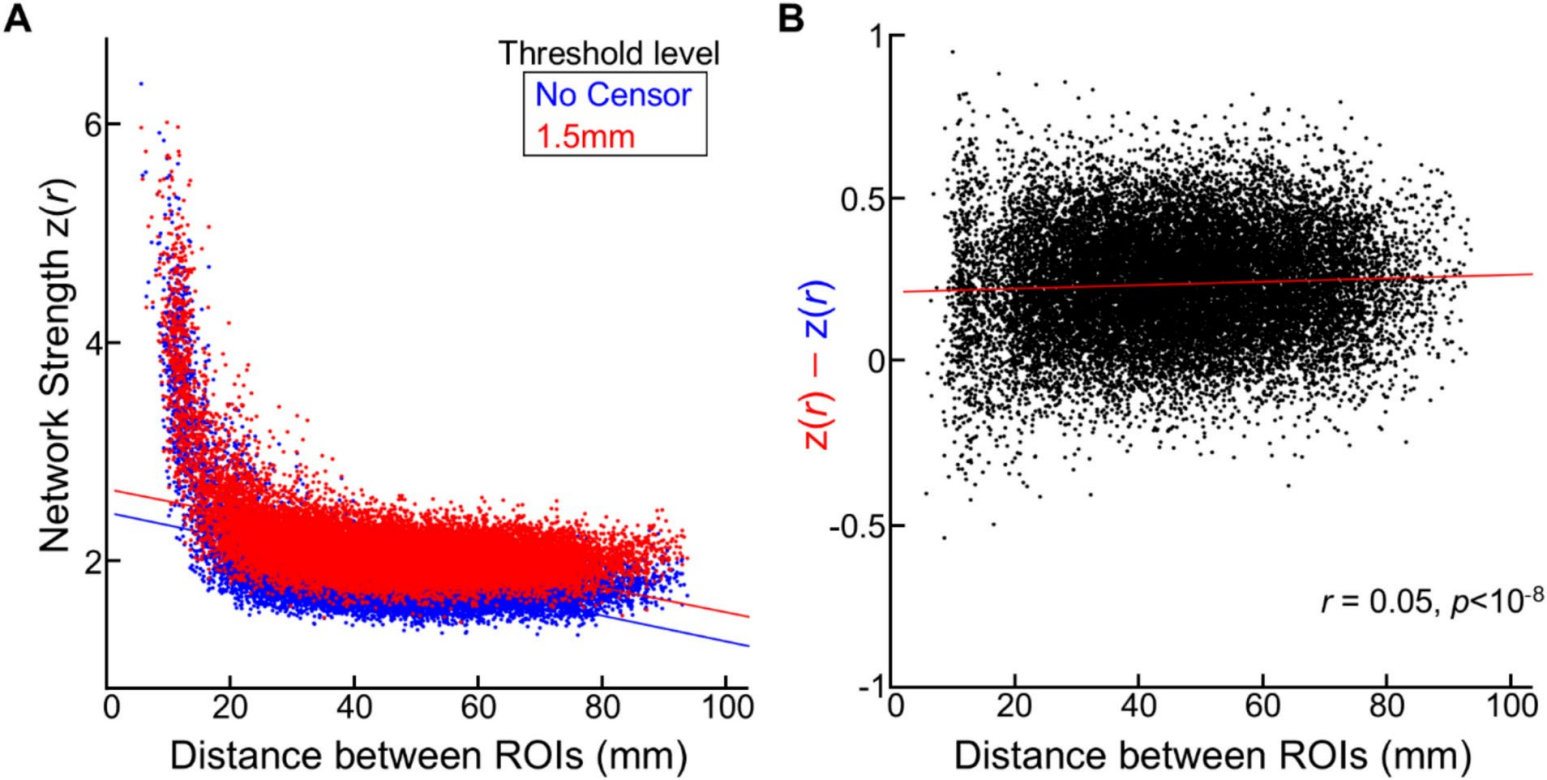
**The influence of head motion on different ranges of functional connectivity.** (**A**) The scatter plot between anatomical distance between parcels and inter-parcel FC strength. Each dot represents the FC (averaged over scans) from all pairs of 200 ROIs. n = 19,900. (**B**) The difference in FC strength between without censoring (colored as red) vs. FD = 1.5 mm (colored as blue). The calculated difference is plotted as a function of distance between ROIs.

In other age groups, such as adults and neonates, long-range functional connectivity (FC) tends to be weaker than short-range FC, and censoring has been shown to alleviate this trend^7,8,12,32^. We examined the change in network strengths over distances in the fetal cohort, using two threshold levels: 1.5 mm and no censoring. Consistent with findings from other age groups, we observed that FC strength between ROIs was negatively associated with the distance between ROIs (Fig. 6A). This trend was evident under both censoring conditions: 1.5 mm (*r* = − 0.46, *p* < 10^–6^) and no censoring (*r* = − 0.52, *p* < 10^–6^). Additionally, we found that thresholding the data at 1.5 mm enhanced overall strength across all distances compared to no censoring, with the increase in strength being more pronounced for longer-range FC (Fig. 6B; *r* = 0.05, *p* < 10^–6^). In summary, while the nuisance regression effectively mitigated the influence of head motion on FCs with appropriate statistical corrections such as FDR correction, a residual motion-related effect on individual FCs remained when no censoring strategy was applied.

### Lingering influence of head motion on FC profile

**Fig. 7:**
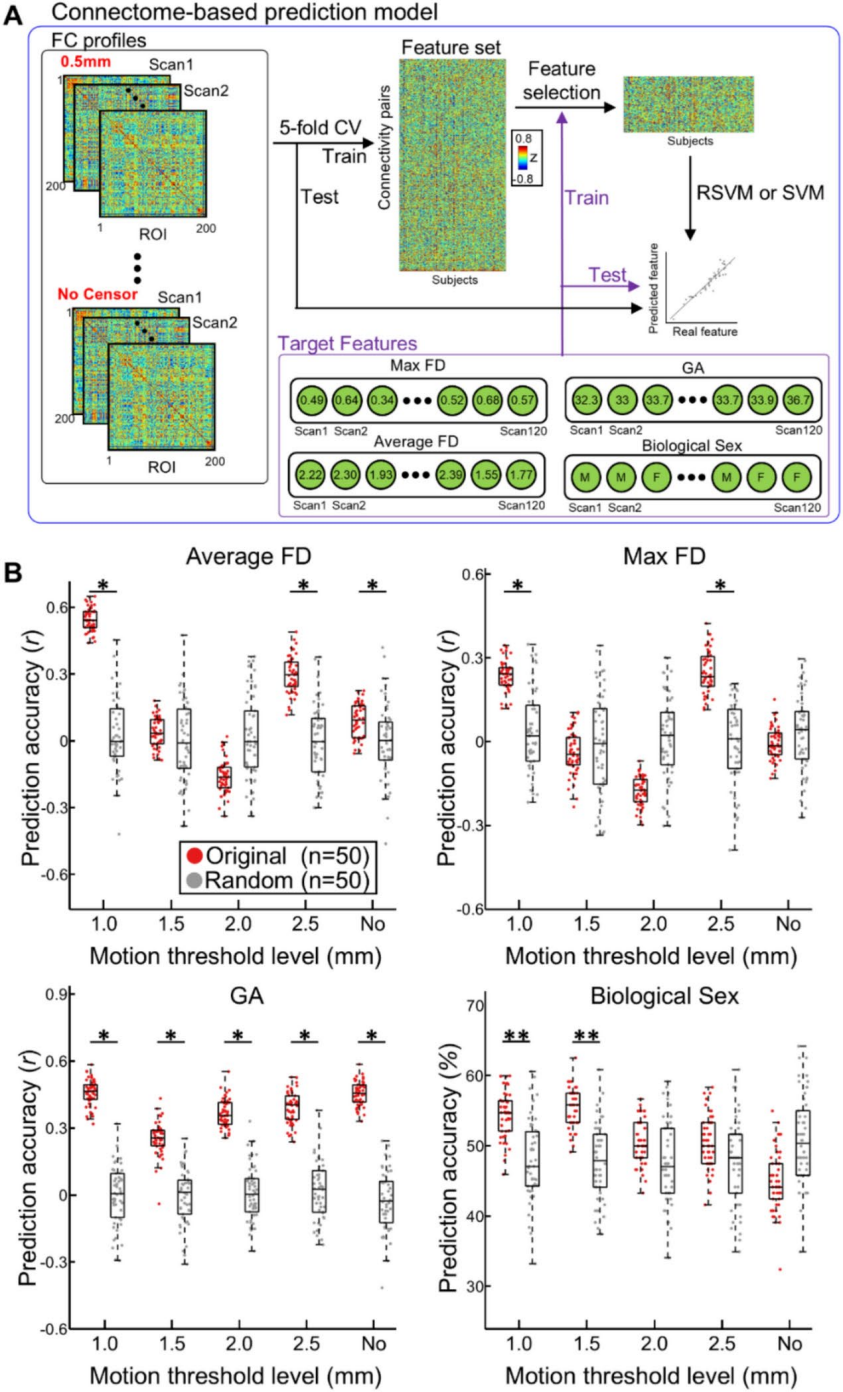
**Functional connectome-based prediction model for FD, GA, and biological sex.** (**A**) Illustration of FC-based prediction scheme. (**B**) Boxplot of prediction accuracy (correlation coefficient for average FD, max FD, GA, and biological sex). Random stands for the prediction task with randomly assigned inputs (e.g., GAs or motion degrees). *: Bonferroni-corrected *p* < 0.05, **: Bonferroni-corrected p < 0.01, one-tailed two-sample t-test.

As the FC profile represents the global pattern of functional brain connectome at the whole brain scale, the impact of head motion on the FC profile can arise even though it was not observable at the level of individual FC. We examined the lingering effect of head motion on fetal FC profile, employing censoring at various threshold levels. Additionally, we investigated whether and, if so, to what extent the censored rs-fMRI data carried neurobiological information, i.e., gestational age (GA) and biological sex. For this purpose, we built a connectome-based prediction model (Fig. 7A). Briefly, we utilized SVM for the prediction task and validated the SVM model’s performance using five-fold cross-validation.

When we applied censoring strategies to the rs-fMRI data, the FC profiles could not predict FD_ave_, except at censoring threshold levels of 1.0 and 2.5 mm (Fig. 7B top left). Similarly, the FC profiles significantly predicted FD_max_ for censoring threshold levels of 1.0 and 2.5 mm (Fig. 7B top right). In contrast, for uncensored rs-fMRI data, the FC profiles of scans were highly predictive of the FD_ave_ levels of scans, indicating the remaining influence of head motion. The FC profile significantly predicted GA for all censoring threshold levels or without censoring (Fig. 7B bottom left). However, only the censored FC profiles, especially within the range of 1.0–1.5 mm censoring threshold levels, showed a successful prediction of sex (Fig. 7B bottom right). Interestingly, when the threshold level was set to 1.5 mm, most of cross-validation trial consistently showed prediction accuracies above the chance level (= 50%). Our findings demonstrated that uncensored or weakly thresholded (e.g., 2.5 mm) FC profiles were still significantly associated with head motion. Censoring within the 1.0–1.5 mm threshold revealed that rs-fMRI data carried the most information about biological sex, resulting in the highest prediction accuracy (54.7 ± 2.8% with 1.5 mm vs. 44.6 ± 3.6% with no censoring). Across different threshold levels, the number of FCs used for GA prediction (survived after the feature selection step) remained largely consistent (6.2, 6.0, 6.4, 5.7, and 6.0% from 1.0 mm to no censoring). At more lenient threshold levels, fewer connections were used for predicting biological sex (4.1 and 4.3% with FD = 1.0 and 1.5 mm vs. 3.6 and 3.3 with FD = 2.5 mm or no censoring). Altogether, this suggests that implementing an appropriate censoring strategy can enhance the reliability of fetal rs-fMRI data, ensuring a more accurate representation of neurobiological information.

### The lingering influence of head motion across different nuisance regression models

**Fig. 8:**
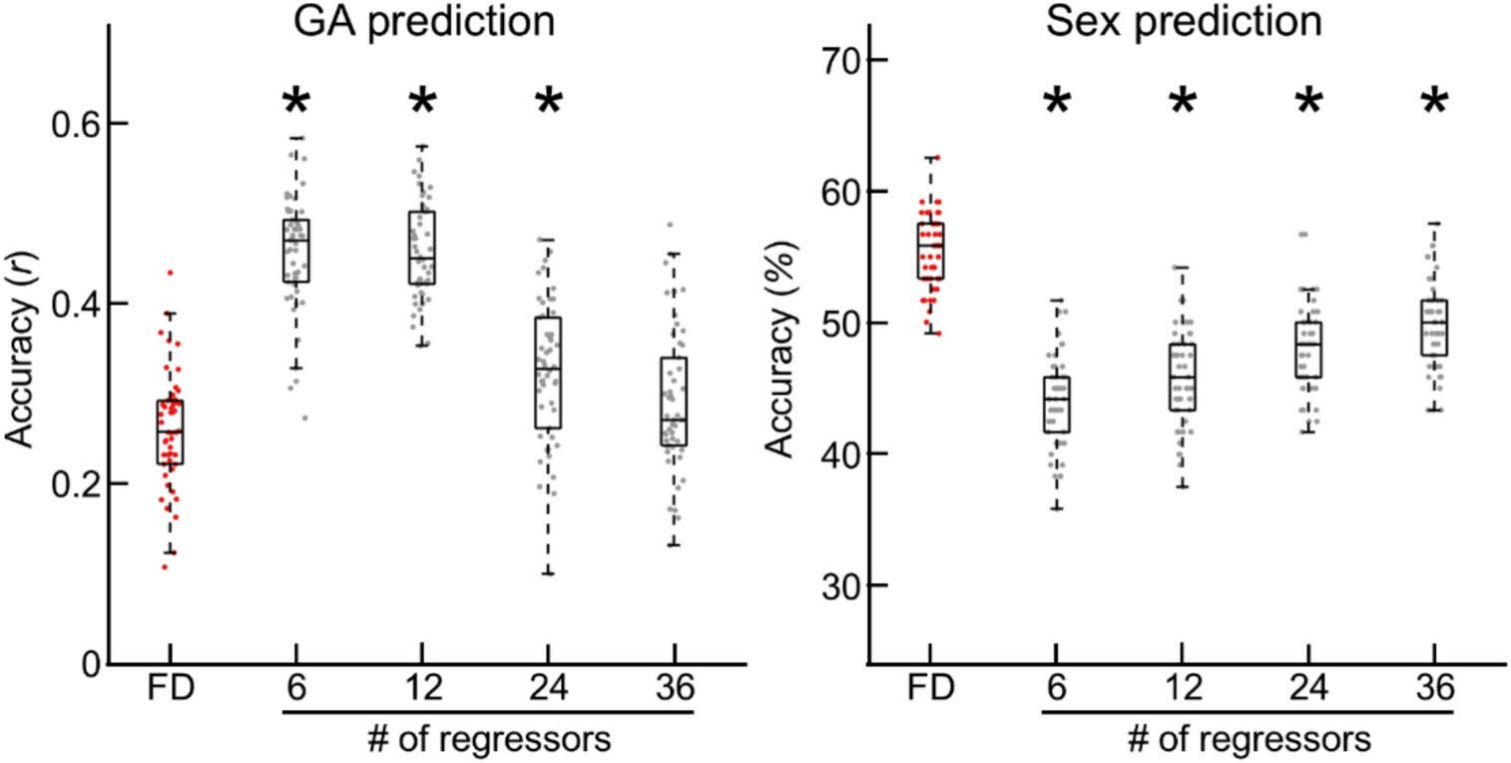
**Prediction accuracy for GA and biological sex across different nuisance regression models.** Boxplot of prediction accuracy for GA (left) and biological sex (right), with data censored at FD = 1.5 mm or data regressed with different sets of regressors. *: Bonferroni-corrected *p* < 10^–4^, two-sample t-test compared to FD.

We further investigated whether the influence of head motion persisted even with more complex regressors, including using different numbers of regressors: 6, 12 (default), 24, and 36. After regressing out fMRI volumes with these varying sets of regressors, we conducted prediction tasks using the regressed fMRI volumes (Fig. 8). For GA, we observed that fMRI data with a higher number of regressors resulted in lower prediction accuracy (Fig. 8 left). For biological sex, there was a slight improvement in prediction accuracy as the number of regressors increased, but it was still significantly worse than the accuracy achieved with censored data (Fig. 8 right; *p* < 10^–4^ for all, two-sample t-test). We speculate that the worse prediction accuracy of sex over increasing number of regressors was partly due to the lower degrees of freedom with a higher number of regressors, as our fetal fMRI data consisted of fewer volumes, between 100 and 140. Overall, these results further support our previous argument regarding the importance of a censoring strategy for improving the reliability of fetal resting-state fMRI data.

### The lingering influence of head motion on the whole brain-scale functional network

**Fig. 9:**
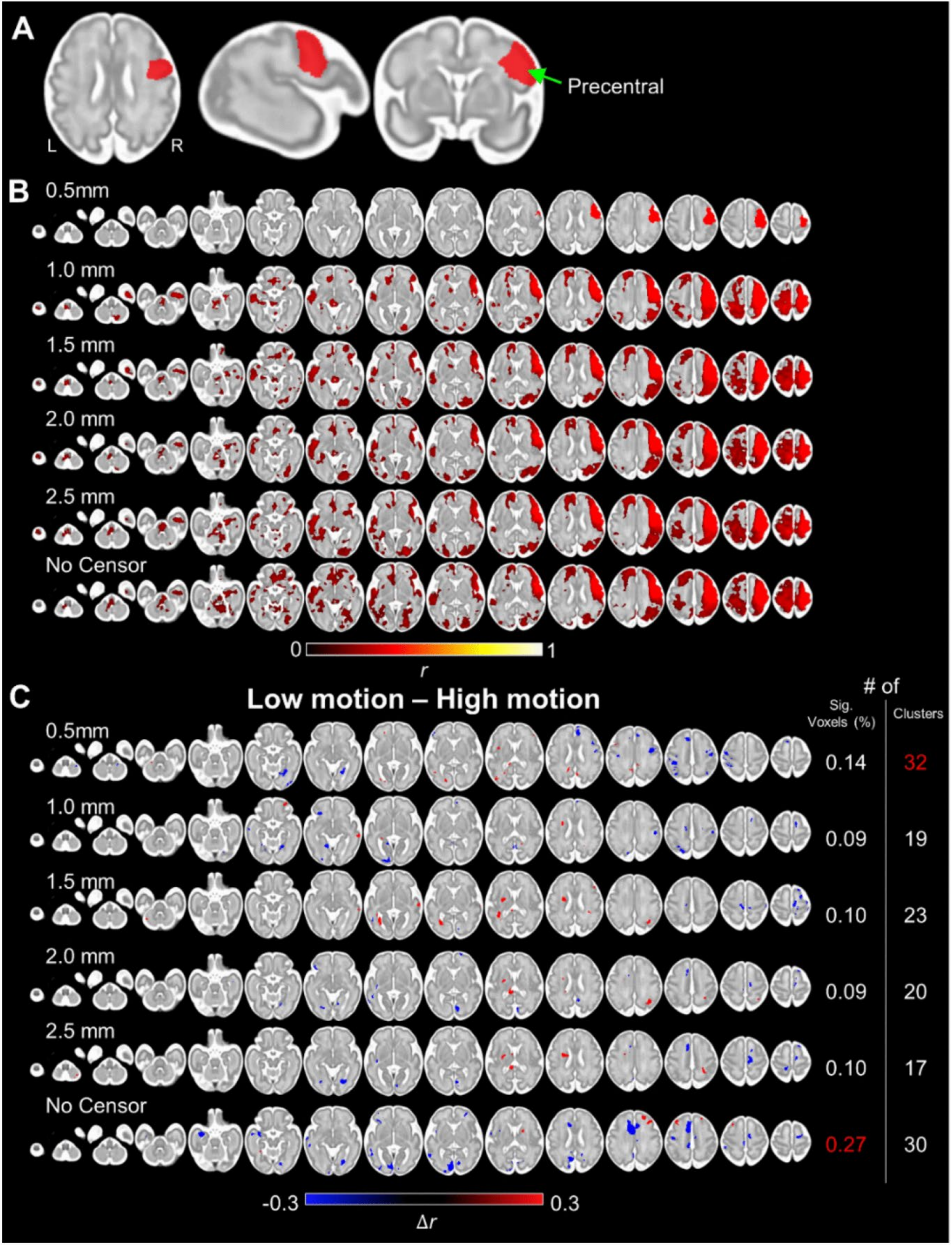
**The influence of head motion on large-scale motor network.** (**A**) Seed region of motor networks used for the seed-based correlation analysis. (**B**) The motor network over different censoring threshold levels. (**C**) Network patterns between higher and lower motion groups with various censoring strategies. The right panel shows the percentage of significant voxels and the number of separate clusters, for each threshold level. The highest number is highlighted in red color. The statistical test uses the cluster-based threshold (uncorrected *p* < 0.01 and > 40 neighboring voxels).

Here, we examined the effectiveness of censoring volumes at the level of the whole-brain network using seed-based correlational analysis. To rule out the possibility that the effect of head motion was due to our choice of brain parcellation, we performed the analysis at the voxel level. As previous fMRI studies^4–6^ suggest that the motor network is relatively well developed in fetuses, we focused on the motor network. Here the right precentral gyrus was used as the motor seed region (Fig. 9A). At the 0.5 mm threshold, we observed the significant network strength only around the seed region. However, when more lenient threshold levels were applied (1.0–2.5 mm and no censoring), we observed bilateral increase at the precentral region (Fig. 9B). Over various threshold levels, the overall motor network pattern was largely consistent, covering, to name a few, bilateral precentral, bilateral postcentral, bilateral mid frontal, bilateral angular, left mid frontal, left superior temporal, and left mid occipital areas. While the overall pattern was consistent, we found data with no censoring yielded the greatest number of significant voxels (3.36% vs. 0.30, 2.27, 2.82, 2.85, and 3.13 for 0.5, 1.0, 1.5, 2.0, and 2.5 mm, respectively). By comparing maps from lower- and higher-motion subgroups (each group consisted of 10% of whole subjects), we assessed the head-motion-induced effects at the group-wise level (Fig. 9C). For all censoring threshold levels, we observed between-group difference while the size varied over different threshold levels. The censoring strategy effectively reduced the size of the group-wise pattern to 0.09% (censoring between 1.0 mm to 2.5 mm), from 0.27% with no censoring. Notably, extreme threshold level (= 0.5 mm) showed increased effect size (= 0.14%) compared to more lenient threshold levels. Censoring strategies with certain threshold levels at the range of 1.0–2.5 mm also effectively reduced the number of significant clusters, for example, 30 without censoring vs. 17 with threshold level = 2.5 mm. This result may suggest that better sensitivity of findings can be achieved by censoring volumes contaminated by high motion.

### Censoring volumes with different motion-related measure, sDVARS

**Fig. 10:**
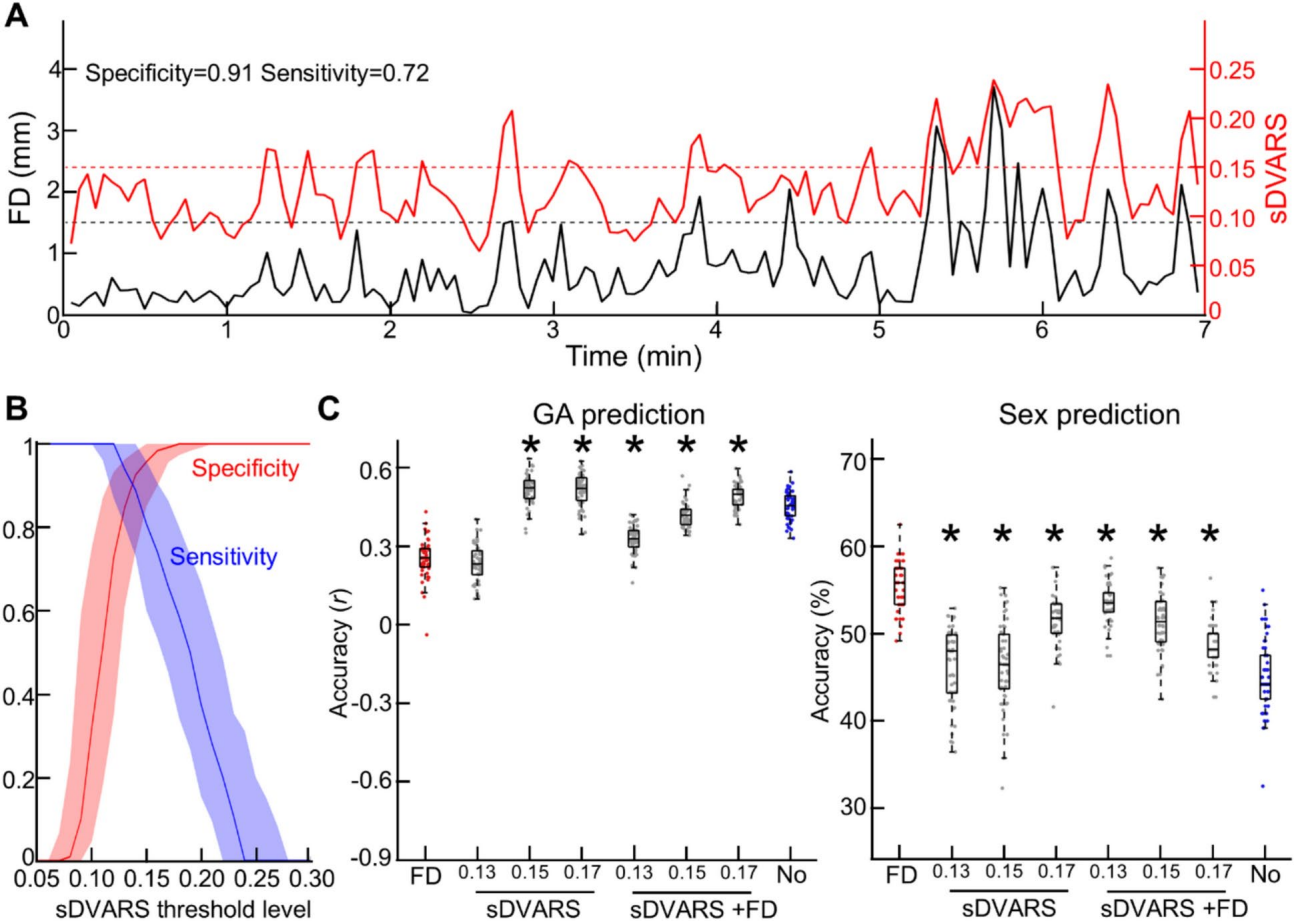
**Censoring volumes with sDVARS.** (**A**) Motion fluctuation during scans in a representative fetus; quantified by frame-wise displacement (FD) and scaled DVARS (sDVARS). (**B**) Median specificity and sensitivity of different sDVARS and FD = 1.5 mm. The shaded area represents the first and third quartiles, respectively. (**C**) Boxplot of prediction accuracy (correlation coefficient for GA and sex). *: Bonferroni-corrected *p* < 0.05, two-sample t-test compared to FD = 1.5 mm (colored as red).

In addition to FD, other metrics are available for assessing the impact of motion on fMRI volumes. Among these, sDVARS—the spatial root mean square of differences between temporally adjacent volumes—is one of the most widely used measures^7,53,54^. In this study, we examined the relationship between FD and sDVARS in the fetal cohort and explored whether sDVARS alone or in combination with FD could produce cleaner fMRI data, as indicated by improved prediction of neurobiological features such as gestational age (GA) and sex. We observed a reasonable concordance between FD and sDVARS; whenever FD showed surges, sDVARS exhibited corresponding increases as well (Fig. 10A). To further quantify the concordance between FD and sDVARS, we calculated sensitivity and specificity across varying sDVARS thresholds relative to FD = 1.5 mm (e.g., specificity = 0.91 and sensitivity = 0.72 at sDVARS = 0.15 for the representative subject shown in Fig. 10A). Our analysis revealed that FD = 1.5 mm corresponded approximately to sDVARS ~ 0.15, with a trade-off between sensitivity (median = 0.96, Q1 = 0.85, Q3 = 1) and specificity (median = 0.81, Q1 = 0.60, Q3 = 0.96) (Fig. 10B). Based on these findings, we censored fMRI volumes using sDVARS thresholds of 0.13, 0.15, and 0.17 and assessed the prediction of gestational age (GA) and biological sex using the censored data (Fig. 10C). We observed that thresholds of sDVARS = 0.15 or 0.17 produced better GA prediction accuracies (*r* = 0.52 ± 0.06 and 0.52 ± 0.06, respectively; *p* < 10^–6^ for both) compared to FD, whereas sDVARS = 0.13 showed no significant improvement (r = 0.23 ± 0.07; *p* = 0.18). However, despite the superior performance in predicting GA, sDVARS resulted in lower prediction accuracy for sex across all thresholds tested (accuracy = 46.3 ± 4.4, 46.3 ± 4.9, and 51.5 ± 2.9; *p* < 10^–6^ for sDVARS = 0.13, 0.15, and 0.17, respectively) compared to FD. When censoring was performed by combining FD (1.5 mm) and sDVARS, the resulting accuracy fell between those obtained with FD and sDVARS individually. This suggests that there were no complementary effects between FD and sDVARS, at least in the studied fetal cohort. However, caution is warranted when interpreting these findings, as we did not conduct an extensive search for the optimal sDVARS threshold, unlike FD. Therefore, our results do not necessarily imply that FD is a superior measure to sDVARS. Nonetheless, the findings indicate that a well-chosen FD threshold can perform comparably to sDVARS, further reinforcing the importance of motion-based censoring strategies for fetal fMRI scans.

## Discussion

Using a large dataset of 120 fetal rs-fMRI scans, we systematically investigated the effectiveness of regression and volume censoring in reducing the impact of head motion on fetal functional connectivity. We found that the conventional nuisance regression approach effectively minimized associations between fetal head motion and region-wise fMRI fluctuations, and between head motion and pairwise functional connectivity (Fig. 4, Tables 3 and 4). Despite this improvement, the effects of motion on FC persisted, as evidenced by fetal FC profiles significantly predicting head motion (Fig. 7). We further observed that employing different nuisance regression strategies with varying number of regressors was minimally effective in reducing head motion, as evidenced by lower prediction accuracy of sex with different # of regressors (= 6, 12, 24, and 36) compared to censoring data (Fig. 8). Applying volume censoring alongside regression, as speculated based on work in ex utero populations across the lifespan^7,12,44^, further dissociated motion from FC. With censoring, FC profiles became predictive of biological signals (i.e., sex) instead of noise (i.e., motion). Lastly, the censoring strategy also reduced the impact of head motion on the fetal motor network (Fig. 9). Our findings provide the first evidence of the efficacy of regression combined with the censoring approach in mitigating fetal head motion effects. It should be noted, however, that a very stringent threshold (e.g., 0.5 mm) significantly reduced the size of analyzable samples and likely offset the benefit of data censoring (Fig. 2). A moderate cut-off (e.g., 1.5 or 2.0 mm), on the other hand, minimized the influence of head motion on FC profiles without significantly compromising the length of the data. Notably, the data censored at moderate threshold levels showed the best prediction performance for neurobiological features, such as gestational age and biological sex. Altogether, our study recommends the usage of volume censoring strategy for fetal rs-fMRI study, to acquire reliable findings.

The lingering influence of head motion on fetal FC when using standard regression alone is consistent with findings in other age groups, such as adults^7^ and neonates^12^. Unlike task-based fMRI, in rs-fMRI, there are no behavioral priors to help interpret the dynamics of brain activity. Thus, this type of time series analysis is not commonly performed^55^. Instead, FC between ROIs is widely used in rs-fMRI analysis^56,57^. For example, previous research has shown that the strength of interregional FC increases over GA, and the association between GA and FC is region-specific^1,3,58,59^. In line with observations in other age populations, we also demonstrated that, under lenient statistical thresholds (such as uncorrected *p* < 0.05 or 0.01), some portions of interregional FC were affected by head motion, carrying significant implications for in-utero neuroimaging studies (Table 4). Our findings collectively underscore the importance of appropriate statistical corrections to ensure the reliability of findings in fetal FC.

Fetuses, expectedly, had higher head motion during scans compared to other age groups, with some scans showing extremely high motion peaks (Fig. 1 and Table 1). In fetal scans, there is a large inter-scan variation in head motion measures (Table 1). As a result, the FC profile of uncensored rs-fMRI was contaminated by head motion (Fig. 5). Our findings demonstrated that the choice of the motion threshold level is crucial: an overly stringent threshold (e.g., 0.5 mm) resulted in a drastic reduction in data length, limiting the availability of samples (Fig. 1), while an overly lenient threshold (e.g., 2.5 mm) left the lingering effects of head motion post-regression unaddressed (Fig. 5). We found that the best prediction accuracy for age and biological sex was between 1.0–1.5 mm, and the rs-fMRI censored at the level of 1.5 mm was not predictive of average and max FD (Fig. 7; 1.5, and 2.0 mm for average FD; 1.5, and 2.0 mm for max FD). Furthermore, censoring effectively reduced head motion’s influence on large-scale brain networks such as the motor network (Fig. 9). Censoring was also effective in reducing the dependency of FC strength on ROI-wise distance (Fig. 6). Furthermore, our findings from the additional analysis with sDVARS suggest that determining the optimal threshold level may be more critical than the choice of motion measure (e.g., FD or sDVARS) when analyzing fetal fMRI data (Fig. 10). However, it is essential to consider that a consequence of censoring is data discontinuity. This precludes the use of analyses such as auto-regression^60^, phase synchrony^61–63^, or dynamic FC analysis^64^. Therefore, the choice of which data censoring strategy to employ should depend on the analytical goals of the study. Specifically, for studies involving analyses only at the level of time series or single interregional interactions (e.g., individual FC), one may ignore additional motion correction techniques after nuisance regression but rather select appropriate statistical methods. On the other hand, using data censoring is highly recommended especially if a study is interrogating whole brain patterns of FC, e.g., brain network analysis. We believe that a 1.5–2.0 mm motion threshold level will be a reasonable starting point for most fetal studies. Still, the optimal threshold level may vary depending on the specific dataset and research purposes. Additionally, the utility of volume censoring can be limited to specific behavioral states or neurological groups, especially if between-group motion differs. Since the brain states of fetuses (e.g., awake, resting, or sleep) were neither identified nor controlled for, it is possible that volume censoring tends to remove volumes associated with an active brain state (i.e., awake) or a more active subject, as those volumes will likely carry higher head motion. Notably, given the unknown brain states of a fetus during a scan, teasing apart the useful brain-state-related signal from motion-related artifact will be one of the bigger challenges for fetal rs-fMRI. Developing more advanced motion-correction algorithms tailored for fetal fMRI would greatly aid in addressing this challenge. Lastly, employing multiple metrics to quantify the degree of head motion, such as root-mean-square (RMS) or Euler Angle (EA), can be useful for accurate identification of motion-contaminated volumes.

As an alternative to data censoring, data-driven denoising algorithms, such as independent component analysis (ICA)-based methods like ICA-AROMA^15^, could be a promising technique for mitigating the effects of head motion on fetal fMRI. One key strength of these denoising algorithms is that they guarantee data continuity (especially with longer data lengths). However, several technical challenges exist, particularly when applied to fetal fMRI data. For effective denoising, ICA-based algorithms require a training phase to identify motion-related or artifact-driven independent components (ICs). This necessitates large sample sizes, which are often difficult to obtain in fetal fMRI studies. From a technical perspective, ICA-based denoising algorithms also require a solid understanding of the components of the ICA, such as distinguishing between neurological activity and non-neurological activities, e.g., noise, respiratory artifacts, and motion-related artifacts. Alternatively, ICA-based denoising algorithms can be applied at the individual-level — manually identifying/removing artifact-like components then reconstructing artifact-free fetal fMRI data. Yet, similar to other automated ICA-based denoising algorithms, manual correction will also necessitate good understanding about spatiotemporal characteristics of neuronal components (e.g., brain network patterns) of fetuses across various gestational ages. While ICA-based denoising algorithms present an appealing alternative to data censoring, they also come with their own set of advantages and challenges compared to censoring. For instance, in our motion study conducted with a neonatal cohort, we observed lingering effects of head motion even after applying ICA-based denoising algorithms^12^. Thus, while promising, ICA-based denoising methods are not without limitations, particularly in fetal fMRI analysis.

Our study has three notable limitations. Firstly, although we evaluated the neurobiological aspects of the data censoring strategy by examining the prediction performance of two features, GA and biological sex, it remains uncertain whether the optimal censoring level for GA and sex prediction can be generalizable across other factors, such as motor or sensory development. We acknowledge that future studies are needed to confirm the appropriateness of the censoring levels (1.5–2.0 mm) over other analyses. Second, we employed the conventional prediction model, SVM, to assess the impact of head motion on neurobiological features. Although SVM is known to be a powerful tool for diverse applications, it is possible that even with uncensored data, significant prediction performance of neurobiological features could be achieved by using more sophisticated prediction models, such as deep learning^65,66^. Additionally, more advanced machine learning algorithms that mitigate the effects of multicollinearity within the feature set can be employed. Nonetheless, we emphasize that our study is a proof-of-concept study that supports using a data censoring strategy in fetal rs-fMRI studies, demonstrating the systematic influence of head motion on uncensored rs-fMRI. Lastly, it is uncertain whether our findings would be generalizable across different fetal rs-fMRI datasets with varying recording environments and preprocessing steps. While it needs confirmation, based on the inter-center generalizability proven in our neonate head motion study^12^, we speculate that the systematic influence of head motion in other fetal datasets should be expected. In contrast, the efficacy of the censoring strategy or the optimal threshold level may vary in different datasets. Confirmation of our findings in diverse fetal rs-fMRI datasets is crucial to establish the reliability and general applicability of data censoring as a potential solution to address head motion impact in fetal neuroimaging studies.

In this study, we provided strong evidence of the significant influence of head motion on resting-state fMRI in fetuses. We found that, with the conventional nuisance regression method commonly used in rs-fMRI analysis, the confounding effect of head motion was effectively mitigated at the level of region-wise fMRI fluctuations or individual FC, along with appropriate statistical tests. However, the pattern of FC profiles was found to be significantly predictive of head motion, indicating a systematic effect of head motion in fetal rs-fMRI, similar to what has been observed in other age groups. To address this systematic influence, we demonstrated that data censoring was highly effective in attenuating the impact of head motion on fetal rs-fMRI. As future work, we would like to compare the novel findings obtained from this study to other pre-existing preprocessing pipelines such as RS-FetMRI^25^, to examine the robustness of our current pipeline. To conclude, we recommend combining regression and censoring techniques in fetal rs-fMRI to reduce potential bias introduced by head motion and improve the reliability of the findings.

## Supplementary Information

Supplementary Information.

## Data Availability

The code used for the analysis is available from the corresponding author, CL, upon reasonable request.
