# Supplementary material for: Evaluating the effects of volume censoring on fetal functional connectivity
Source: Sci Rep. 2025 Apr 16;15:13181. doi: 10.1038/s41598-025-96538-x (PMC12003846; doi:10.1038/s41598-025-96538-x)
Supplement: Supplementary file 1 — Supplementary Information. [file 41598_2025_96538_MOESM1_ESM.docx]

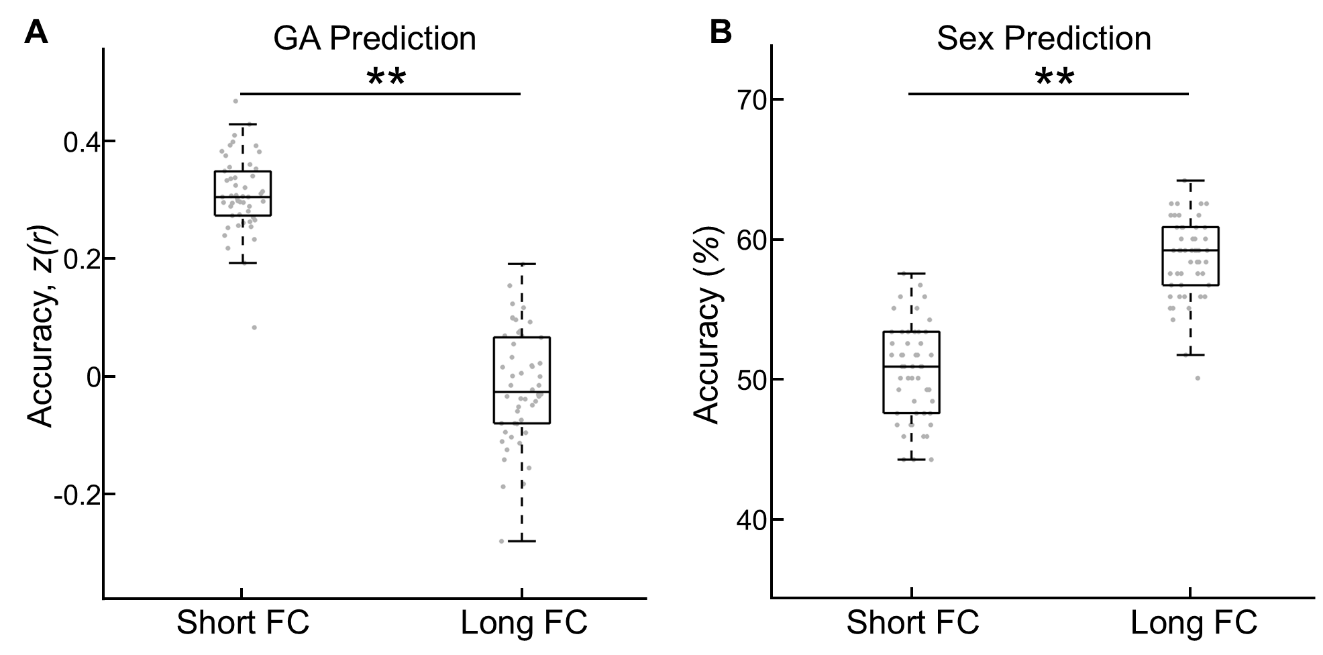


**Supplementary Figure 1.** **Difference of** **Prediction accuracy between long and short FCs.** Boxplot of prediction accuracy for GA (**A**) and biological sex (**B**). **: Bonferroni-corrected *p*<10^-4^, two-sample t-test.
